# FOXO1 Is a Key Mediator of Glucocorticoid-Induced Expression of Tristetraprolin in MDA-MB-231 Breast Cancer Cells

**DOI:** 10.3390/ijms232213673

**Published:** 2022-11-08

**Authors:** Do Yong Jeon, So Yeon Jeong, Ju Won Lee, Jeonghwan Kim, Jee Hyun Kim, Hun Su Chu, Won Jin Jeong, Byung Ju Lee, Byungyong Ahn, Junil Kim, Seong Hee Choi, Jeong Woo Park

**Affiliations:** 1 Department of Biological Sciences, University of Ulsan, Ulsan 44610, Korea; 2 School of System Biomedical Science, Soongsil University, Seoul 06978, Korea; 3 RopheLBio, B102, Seoul Forest M Tower, Seoul 04778, Korea; 4 Department of Food Science and Nutrition, University of Ulsan, Ulsan 44610, Korea

**Keywords:** tristetraprolin, FOXO1, dexamethasone, betamethasone, cancer cells

## Abstract

The mRNA destabilizing factor tristetraprolin (TTP) functions as a tumor suppressor by down-regulating cancer-associated genes. *TTP* expression is significantly reduced in various cancers, which contributes to cancer processes. Enforced expression of *TTP* impairs tumorigenesis and abolishes maintenance of the malignant state, emphasizing the need to identify a *TTP* inducer in cancer cells. To search for novel candidate agents for inducing TTP in cancer cells, we screened a library containing 1019 natural compounds using MCF-7 breast cancer cells transfected with a reporter vector containing the *TTP* promoter upstream of the luciferase gene. We identified one molecule, of which the enantiomers are betamethasone 21-phosphate (BTM-21-P) and dexamethasone 21-phosphate (BTM-21-P), as a potent inducer of *TTP* in cancer cells. We confirmed that BTM-21-P, DXM-21-P, and dexamethasone (DXM) induced the expression of *TTP* in MDA-MB-231 cells in a glucocorticoid receptor (GR)-dependent manner. To identify potential pathways linking BTM-21-P and DXM-21-P to *TTP* induction, we performed an RNA sequencing-based transcriptome analysis of MDA-MB-231 cells at 3 h after treatment with these compounds. A heat map analysis of FPKM expression showed a similar expression pattern between cells treated with the two compounds. The KEGG pathway analysis results revealed that the upregulated DEGs were strongly associated with several pathways, including the Hippo signaling pathway, PI3K-Akt signaling pathway, FOXO signaling pathway, NF-κB signaling pathway, and p53 signaling pathway. Inhibition of the FOXO pathway using a FOXO1 inhibitor blocked the effects of BTM-21-P and DXM-21-P on the induction of *TTP* in MDA-MB-231 cells. We found that DXM enhanced the binding of FOXO1 to the *TTP* promoter in a GR-dependent manner. In conclusion, we identified a natural compound of which the enantiomers are DXM-21-P and BTM-21-P as a potent inducer of *TTP* in breast cancer cells. We also present new insights into the role of FOXO1 in the DXM-21-P- and BTM-21-P-induced expression of *TTP* in cancer cells.

## 1. Introduction

Tristetraprolin (TTP, ZFP36) is an RNA binding protein that binds to AU-rich elements (AREs) within the 3′-untranslated region (3′-UTR) of mRNAs and promotes the degradation of these mRNAs [1,2]. AREs are found within the 3′-UTR of many short-lived mRNAs, such as cytokines and proto-oncogenes mRNAs [3]. TTP functions as a tumor suppressor by destabilizing the mRNA of critical genes implicated in both tumor onset and progression [4,5]. *TTP* is widely expressed, with particularly high levels in spleen, lymph nodes, and thymus [6]. However, *TTP* expression is significantly decreased in various cancers [7]; its downregulation correlates with increased expression of proto-oncogenes and may contribute to cancer processes. Re-expression of TTP in cancer cells has a growth inhibitory effect [8,9,10]. The expression of *TTP* in cancer cells is induced by p53 [11] and inhibited by Myc [12]. Notably, nearly all types of cancers have abnormalities in the p53 pathway [13], and c-Myc is often activated in human cancers [14]. Together, these features may lead to a widespread decrease in the expression of *TTP* in human cancers. Thus, enforced expression of *TTP* may represent a new therapeutic avenue for cancer prevention and treatment.

Previously it has been reported that glucocorticoids (GCs) induce the expression of *TTP* in cells [15,16,17,18]. However, the mechanisms underlying the GC-mediated TTP induction in cancer cells remain unclear. GCs are steroid hormones synthesized and released by the adrenal glands in response to physiological cues and stress [19]. GCs regulate fundamental body functions in mammals, including control of cell growth, development, metabolic homeostasis, cognition, mental health, immune homeostasis, and apoptosis [20,21,22,23]. Various synthetic GCs (e.g., prednisolone, aldosterone, dexamethasone, and betamethasone) have been developed by the pharmaceutical industry and serve as treatments for various diseases. Both natural and synthetic glucocorticoid hormones exert their biological effects predominantly via the glucocorticoid receptor (GR; NR3C1) [24], a ligand-activated transcription factor that is constitutively and ubiquitously expressed throughout the body [25]. GR functions by regulating the expression of GC responsive genes in a positive or negative manner. Upon ligand binding, GR shuttles into the nucleus [22] and binds to DNA sequences called glucocorticoid response elements (GREs) as a homodimeric complex [26]. The binding of GR homodimers to GRE sequences leads to an enhancement of gene expression [27]. GR also interacts with DNA as a monomer by binding to GRE half-sites and positively or negatively influences the transcription of target genes by interacting with promoter-bound STAT5, activator protein 1 (AP-1), or NF-κB transcription factors [28,29]. Independent of binding to GRE, monomeric GR can also regulate gene expression in a mechanism known as tethering, which involves physical interaction of monomeric GR with another transcription factor, such as AP-1 and NF-κB [30]. GCs also exert rapid non-genomic effects that do not require transcription processes or protein synthesis [31]. Interestingly, GCs not only increase the expression of *FOXO* [32,33,34] but also increase FOXO activity [35,36] in a variety cells.

The FOXO family of transcription factors comprises four closely related members—FOXO1, FOXO3, FOXO4, and FOXO6—that are direct downstream targets of AKT [37,38]. FOXOs play central roles in regulating normal hematopoiesis and are integral mediators of AKT actions in cellular growth and survival [38,39]. In the absence of active AKT, FOXOs localize to the nucleus, where they regulate the transcription of genes involved in cell-cycle arrest, apoptosis, and reactive oxygen species (ROS) detoxification. Previous studies have reported that GCs increase *FOXO1* level in a variety of cells, including hepatocytes, cardiomyocytes, and tenocytes [32,33,34]. A reduction in FOXO1 protein protects against beta cell death induced by the synthetic GC dexamethasone, suggesting that FOXO1 activation mediates the pro-apoptotic effects of GCs [40].

Several compounds and cytokines have been identified to induce *TTP* expression [4,5]. However, until now, there has been no report regarding the identification of compounds from library screening. The purpose of this study was to identify natural compounds that induce the expression of *TTP* in cancer cells by screening a natural compound library using a cell-based reporter assay. Among the 1019 natural compounds in the library, we identified one molecule, of which the enantiomers are betamethasone 21-phosphate (BTM-21-P) and dexamethasone 21-phosphate (DXM-21-P), as the best inducer of *TTP* in the cell-based reporter assay. We show here, for the first time, that GCs such as BTM-21-P, DXM-21-P, and DXM induce the expression of *TTP* in a FOXO1-dependent manner, even in p53 mutant breast cancer cells. The inhibition of GR or FOXO1 by inhibitors abrogated the effects of GCs with respect to *TTP* induction. We also found that GC-induced *TTP* is required for the growth inhibitory effect of GC on breast cancer cells. Together, these studies identify a novel signaling pathway by which GCs induce *TTP* expression in a FOXO1-dependent manner, representing a possible novel pharmacological approach to treat p53 mutant breast cancer cells.

## 2. Results

### 2.1. Screening of a Natural Compound Library Identified an Enantiomer of Betamethasone 21-Phosphate and Dexamethasone 21-Phosphate as a Potent Inducer of TTP in Cancer Cells

**Figure 1:**
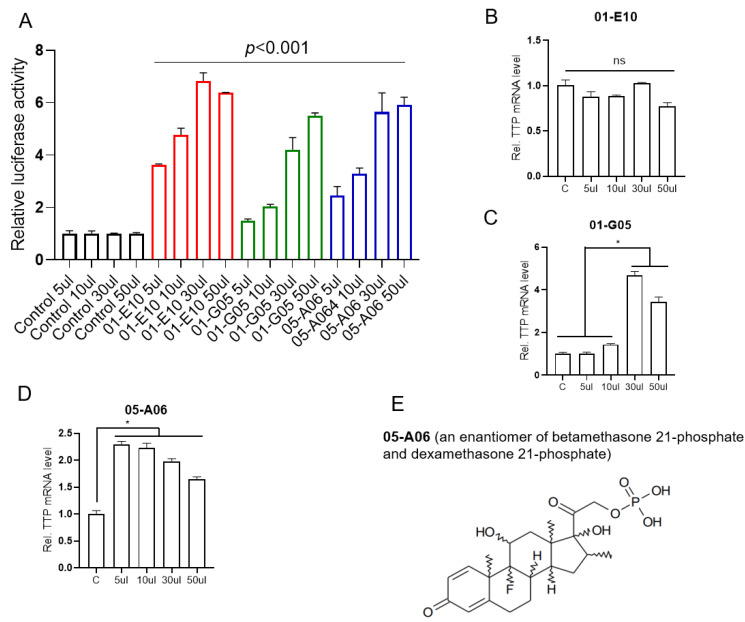
Dose-dependent induction of *TTP* in MDA-MB-231 cells by three compounds selected from the fourth screening of the natural compound library. (**A**) Dose-dependent changes in luciferase activity. MDA-MB-231 cells were transfected with pGL3/TTPp-1343 luciferase reporter vector containing the human *TTP* promoter, followed by stimulation with different concentrations of three selected compounds solubilized in DMSO for 48 h. The same volume of DMSO was added to the cells as controls. For each compound, the fold change in luciferase activity of stimulated cells was calculated relative to that of the DMSO control. The graphs are mean ± SD of three independent experiments (Two-way ANOVA). Black bars, control; red bars, 01-E10; green bars, 01-G05; violet bars, 05-A06. (**B**–**D**) Dose-dependent changes in endogenous *TTP* level. MDA-MB-231 cells were stimulated with different concentrations of compounds (**B**) 01-E10, (**C**) 01-G05, and (**D**) 05-A06 for 48 h. The expression level of *TTP* mRNA was analyzed by qRT-PCR. For each compound, the fold change in expression level was calculated relative to that of the DMSO control. The graphs are mean ± SD of three independent experiments (one-way ANOVA, * *p* < 0.01). (**E**) Structure of compound 05-A06, an enantiomer of BTM-21-P and DXM-21-P.

To perform cell-based screening for compounds that induce *TTP* expression in breast cancer cells, we used a library containing 1019 natural compounds and MCF-7 cells transiently transfected with the pGL3/TTPp-1343 construct containing the *TTP* promoter upstream of a luciferase reporter gene. Each natural compound was added to the transfected cells, and the effect of the compound on *TTP* induction was measured at 24 h post-treatment by luciferase assay. After three rounds of screening, we identified five compounds that induced a greater than two-fold increase in luciferase activity (Tables S1 and S2). To determine whether the selected compounds enhanced *TTP* promoter activity in p53 mutant breast cancer cells, we conducted luciferase reporter assays with the reporter vector in MDA-MB-231 cells that express mutant p53. Among the five identified compounds, three increased the luciferase activity in a dose-dependent manner (Table S2 and Figure 1A). We next tested these three compounds for their effect on the expression of endogenous *TTP* in MDA-MB-231 cells. Among the three compounds, compound 05-A06 was the most potent inducer of endogenous *TTP* at low volume (5 μL) in MDA-MB-231 cells (Figure 1B–D). At 50 μL, compounds 01-G05 and 05-A06 were toxic to MDA-MB-231 cells, which might lead to a decrease in *TTP* expression (Figure 1C,D). Compound 05-A06 is an enantiomer of betamethasone 21-phosphate (BTM-21-P) and dexamethasone 21-phosphate (DXM-21-P) (Figure 1E).

### 2.2. Betamethasone 21-Phosphate, Dexamethasone 21-Phosphate, and Dexamethasone Induce the Expression of TTP in MDA-MB-231 Cells

**Figure 2:**
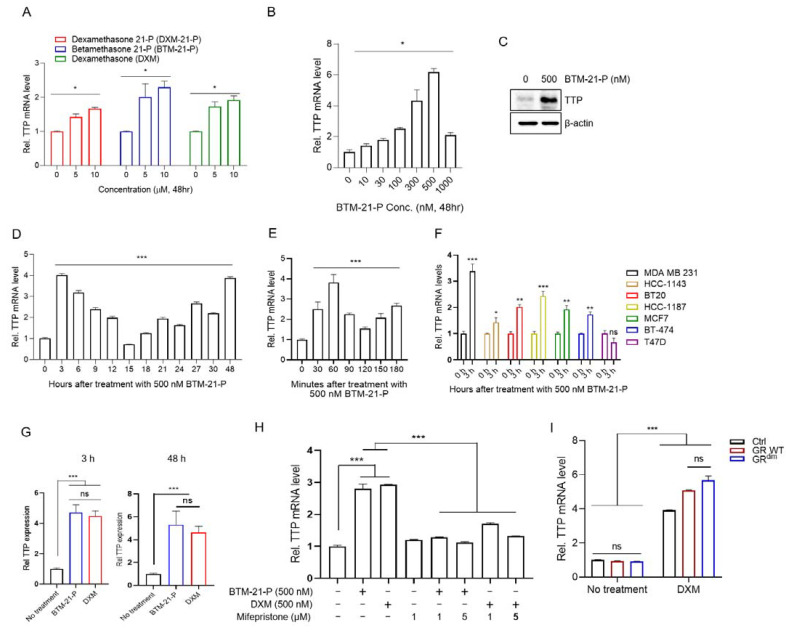
Induction of *TTP* expression by BTM-21-P, DXM-21-P, and DXM in breast cancer cells in a glucocorticoid receptor (GR)-dependent manner. (**A**,**B**) Dose-dependent changes in *TTP* expression level in MDA-MB-231 cells. MDA-MB-231 cells were stimulated with the indicated concentration of BTM-21-P, DXM-21-P, or DXM for 48 h. The expression level of *TTP* was analyzed by qRT-PCR. For each compound, the fold change in expression level was calculated relative to that of the DMSO control. The graphs are mean ± SD of three independent experiments (one-way ANOVA, * *p* < 0.01). (**C**) Western blotting analysis for TTP in MDA-MB-231 cells stimulated by 500 nM BTM-21-P for 48 h. (**D**,**E**) Time-dependent changes in *TTP* expression level in MDA-MB-231 cells. MDA-MB-231 cells were stimulated with 500 nM BTM-21-P for the indicated times. The expression level of *TTP* mRNA was analyzed by qRT-PCR. Fold change in expression level was calculated relative to that of the DMSO control. The graphs are mean ± SD of three independent experiments (one-way ANOVA, *** *p* < 0.001). (**F**) Effects of BTM-21-P on *TTP* expression in several breast cancer cell lines (MDA-MB-231, HCC-1143, BT20, HCC-1187, MCF-7, BT-474, and T47D) stimulated with 500 nM BTM-21-P for 3 h. The expression level of *TTP* was analyzed by qRT-PCR. Fold change in expression level was calculated relative to that of the DMSO control. The graphs are mean ± SD of three independent experiments (Student’s *t* test, * *p* < 0.01; ** *p* < 0.005; *** *p* < 0.001). ns, not significant. (**G**) Comparison of the effects of BTM-21-P and DXM on *TTP* expression in MDA-MB-231 cells. MDA-MB-231 cells were stimulated with 500 nM BTM-21-P or 500 nM DXM for 3 h or 48 h. The expression level of *TTP* mRNA was analyzed by qRT-PCR. Fold change in expression level was calculated relative to that of the DMSO control. The graphs are mean ± SD of three independent experiments (Student’s *t* test, *** *p* < 0.001). ns, not significant. Blue bars, BTM-21-P; red bars, DXM. (**H**) The effect of the GR inhibitor on the BTM-21-P- and DXM-mediated induction of *TTP* expression in MDA-MB-231 cells. MDA-MB-231 cells were treated with indicated concentrations of the GR inhibitor mifepristone for 30 min, followed by stimulation with 500 nM BTM-21-P or 500 nM DXM for 3 h. The expression level of *TTP* mRNA was analyzed by qRT-PCR. Fold change in expression level was calculated relative to that of the DMSO control. The graphs are mean ± SD of three independent experiment (one-way ANOVA, *** *p* < 0.001). (**I**) The effect of dimerization-defective GR (GR^dim^) on the DXM-mediated induction of *TTP* expression in MDA-MB-231 cells. MDA-MB-231 cells were transfected with empty vector, pGR, or pGRdim, followed by 500 nM DXM for 3 h. The expression level of *TTP* mRNA was analyzed by qRT-PCR. Fold change in expression level was calculated relative to that of the DMSO control. The graphs are mean ± SD of three independent experiments (two-way ANOVA, *** *p* < 0.001). ns, not significant.

We next tested whether BTM-21-P and DXM-21-P, enantiomers of the identified compound, induced the expression of endogenous *TTP* in MDA-MB-231 TNBC cells. DXM-21-P is a prodrug that is converted to DXM. We also used dexamethasone (DXM) to compare its *TTP*-inducing ability with those of BTM-21-P and DXM-21-P. All three compounds induced the expression of endogenous *TTP* in MDA-MB-231 cells at 24 h post-treatment (Figure 2A). To determine the optimal concentration for *TTP* induction, MDA-MB-231 cells were treated with various concentrations of BTM-21-P for 48 h, and *TTP* mRNA expression was examined by by qRT-PCR. *TTP* mRNA expression was highest in response to 500 nM of BTM-21-P in MDA-MB-231 cells (Figure 2B). TTP protein level in MDA-MB-231 cells also was increased by treatment with 500 nM of BTM-21-P for 48 h (Figure 2C). We next treated MDA-MB-231 cells with 500 nM BTM-21-P and collected cells from 3 h to 30 h post-treatment at 3-h intervals and at 48 h post-treatment. *TTP* expression level fluctuated with time after BTN-21-P treatment and peaked at 3 h and 48 h post-treatment (Figure 2D). These data suggest that BTM-21-P may induce *TTP* expression earlier than 3 h post-treatment. We thus analyzed *TTP* expression at 30-min intervals after BTM-21-P treatment. As shown in Figure 2E, *TTP* expression level reached a peak at 1 h after BTM-21-P treatment (Figure 2E). We further analyzed the effect of BTM-21-P on *TTP* expression in other breast cancer cell lines such as HCC-1143, BT20, HCC-1187, MCF-7, BT-474, and T47D. BTM-21-P induced *TTP* expression in all breast cancer cell lines except the T47D cell line (Figure 2F). MDA-MB-231 cells treated with 500 nM DXM expressed a similar level of *TTP* as cells treated with 500 nM BTM-21-P at 3 h and 48 h post-treatment (Figure 2G).

GCs exert their biological effects predominantly via GR [24]. Mifepristone is a potent antagonist of progesterone receptors and GR and has been used as an abortifacient. To determine whether BTM-21-P and DXM induce *TTP* expression via GR, we pretreated MDA-MB-231 cells with the GR inhibitor mifepristone and tested the effects of BTM-21-P and DXM on *TTP* expression. The inhibition of GR using mifepristone blocked the BTM-21-P- and DXM-induced expression of *TTP* in a dose-dependent manner in MDA-MB-231 cells (Figure 2H).

GR regulates gene expression as either a homodimer or monomer [27,28,29]. To determine whether GR dimerization is required for TTP induction, we transfected MDA-MB-231 cells with plasmid expressing wild-type GR or dimerization-defective GR (GR^dim^) and analyzed the expression of *TTP* after DXM treatment. In the absence of DXM treatment, ectopic expression of either wild-type GR or GR^dim^ did not induce the expression of *TTP* (Figure 2I). DXM treatment significantly increased *TTP* expression in both wild-type GR– and GR^dim^-transfected cells, and there was no significant difference in *TTP* expression levels (Figure 2I). This result indicates that GR dimerization is not required for *TTP* induction by DXM.

### 2.3. RNA-Seq Transcriptome Analysis of Betamethasone 21-Phosphate and Dexamethasone-Treated MDA-MB-231 Cells

**Figure 3:**
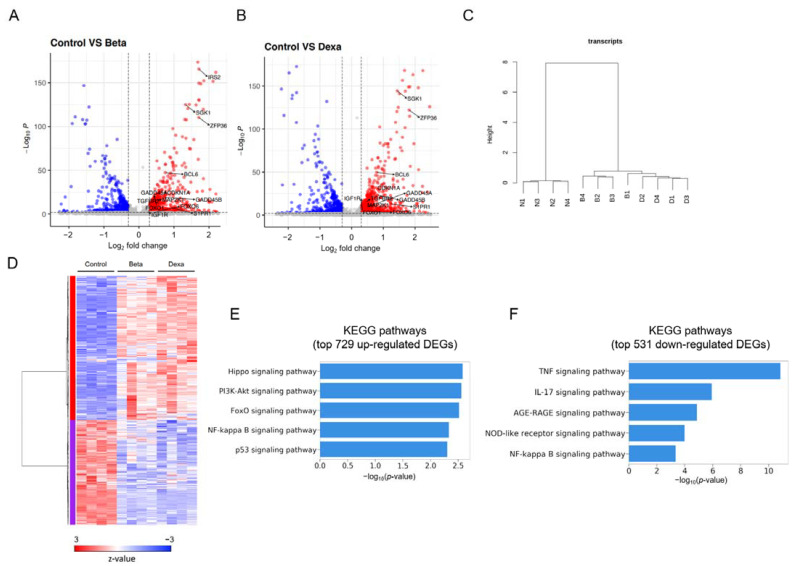
RNA-Seq data analysis and KEGG pathways. (**A**,**B**) Volcano plots for the differentially expressed genes (DEGs) in BTM-21-P-treated and DXM-21-P-treated cells compared with untreated cells. The *y*-axis corresponds to the significance level represented with -log_10_*p* value, and the *x*-axis displays the log_2_ (FC) value. The red dots represent the significant (*p* ≤ 0.01 and |FC| ≥ 0.3) DEGs in (**A**) BTM-21-P-treated cells and (**B**) DXM-21-P-treated cells. The dotted horizontal line means *p* = 0.01, and the dotted vertical lines mean |FC| = 0.3. (**C**) Unsupervised hierarchical clustering of RNA-Seq data from untreated (N1–N4), BTM-21-P-treated (B1–B4), and DXM-21-P-treated (D1–D4) MDA-MB-231 cells. The similarities were calculated by Ward’s criterion for 1-(Pearson’s correlation coefficient). (**D**) Unsupervised hierarchical clustering and heatmap of DEGs clustered into up-regulated and down-regulated DEGs. (**E**,**F**) The *p* values and names of the 5 most over-represented KEGG pathways, calculated on the basis of (**E**) the top 729 up-regulated DEGs and (**F**) the top 531 down-regulated DEGs after treatment with BTM-21-P and DXM-21-P.

To gain insights into the molecular mechanism underlying the DXM-mediated induction of *TTP* in MDA-MB-231 cells, we performed RNA-seq analysis of DXM-treated MDA-MB-231 cells. To determine whether the gene expression profiles of DXM-treated cells were similar to those of BTM-21-P-treated cells, we also performed an RNA-seq analysis of BTM-21-P-treated MDA-MB-231 cells. We collected cells at 3 h post-treatment, as both DXM and BTM-21-P induced TTP expression in MDA-MB-231 cells at this time point. Non-treated cells were used as controls. We determined differential gene expression between non-treated and BTM-21-P- or DXM-treated cells by comparing the three groups using EdgeR [41]. A total of 1260 differentially expressed genes (DEGs) (FDR < 0.01) with an absolute log2fold change of 0.3 or greater was detected in BTM-21-P-treated cells and DXM-treated cells compared with non-treated cells (Table S3). In the comparison of BTM-21-P-treated cells with non-treated cells, 927 DEGs were identified, with 544 up-regulated and 383 down-regulated DEGs, as shown in the volcano plot (Figure 3A). Similarly, in the comparison of DXM-treated cells with non-treated cells, 1107 DEGs were identified, with 655 up-regulated and 452 down-regulated DEGs (Figure 3B). Two-way unsupervised hierarchical clustering of the union of the DEGs showed a clear separation of BTM-21-P-treated cells (B1–B4) and DXM-treated cells (D1–D4) from non-treated cells (N1–N4) (Figure 3C). However, no recognizable clustering pattern from BTM-21-P-treated cells (B1–B4) and DXM-treated cells (D1–D4) was detected in unsupervised hierarchical clustering (Figure 3C), suggesting a high degree of similarity among them. The DEGs were separated into two clusters of 729 up-regulated and 531 down-regulated DEGs, both in BTM-21-P- and DXM-treated cells (Figure 3D). DEGs identified in biological replicates clustered together, indicating good reproducibility of the treatments (Figure 3D). DEGs identified in BTM-21-P-treated cells and DXM-treated cells showed no significant intergroup differences (Figure 3D), suggesting that BTM-21-P and DXM affect similar signaling pathways in MDA-MB-231 cells.

To identify pathways modulated by BTM-21-P and DXM, we analyzed the Kyoto Encyclopedia of Genes and Genomes (KEGG) pathways of 729 upregulated and 531 down-regulated DEGs in BTM-21-P- and DXM-21-P-treated cells. The KEGG pathway analysis results showed that the upregulated DEGs were associated with several signaling pathways, including the Hippo signaling pathway, PI3K-AKT signaling pathway, FOXO signaling pathway, NF-κB signaling pathway, and p53 signaling pathway (Figure 3E). The down-regulated DEGs were associated with the TNF signaling pathway, IL-17 signaling pathway, AGE-RAGE signaling pathway, NOD-like receptor signaling pathway, and NF-κB signaling pathway (Figure 3F).

### 2.4. FOXO1 Mediates Dexamethasone- and Betamethasone 21-Phosphate-Induced TTP Expression in MDA-MB-231 Cells

**Figure 4:**
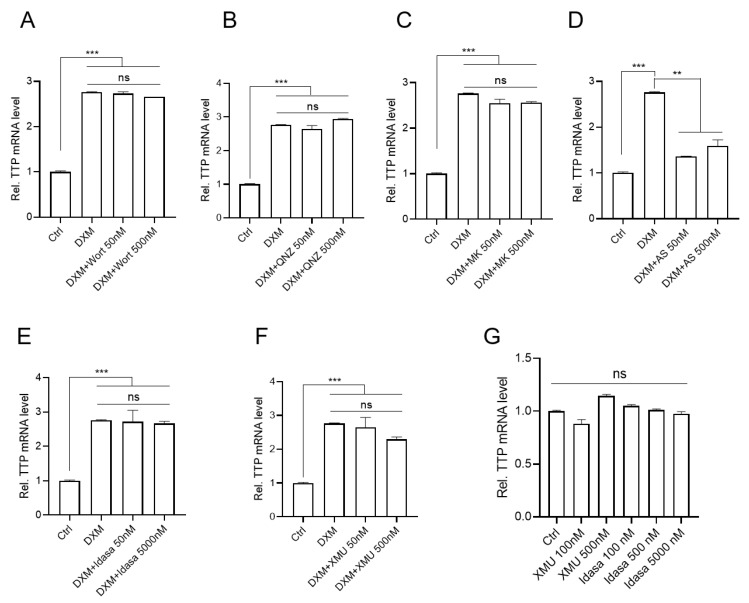
FOXO1 mediates DXM-induced *TTP* expression in MDA-MB-231 cells. (**A**–**F**) MDA-MB-231 cells were incubated with the indicated concentrations of inhibitors against (**A**) PI3K (Wortmannin) (**B**) NF-κB (QNZ), (**C**) AKT (MK2206), (**D**) FOXO1 (AS1842856), (**E**) MDM2 (Idasanutlin), and (**F**) MST1/2 (XMU-MP-1) for 12 h, followed by stimulation with 500 nM DXM for 3 h. The expression level of *TTP* mRNA was analyzed by qRT-PCR. Fold change in expression level was calculated relative to that of the DMSO control. The graphs are mean± SD of three independent experiment (one-way ANOVA, ** *p* < 0.005; *** *p* < 0.001). ns, not significant. (**G**) MDA-MB-231 cells were incubated in the presence of the indicated concentration of inhibitors against MST1/2 (XMU-MP-1) and MDM2 (Idasanutlin) for 12 h. The expression level of *TTP* mRNA was analyzed by qRT-PCR. Fold change in expression level was calculated relative to that of the DMSO control. The graphs are mean ± SD of three independent experiments (one-way ANOVA). ns, not significant.

To further uncover the signal pathways involved in DXM- and BTM-21-P-induced expression of *TTP* in MDA-MB-231 cells, and to determine whether any of the upregulated KEGG pathways mediated the DXM- and BTM-21-P-induced expression of TTP in MDA-MB-231 cells, we analyzed the effects of inhibitors against PI3K (Wortmannin) (Figure 4A and Figure S1A), NF-κB (QNZ) (Figure 4B and Figure S1B), AKT (MK2206) (Figure 4C and Figure S1C), and FOXO1 (AS1842856) (Figure 4D and Figure S1D) on the DXM- and BTM-21-P-induced expression of *TTP* in cancer cells. Only the FOXO1 inhibitor blocked the effects of both DXM and BTM-21-P on the induction of *TTP* in MDA-MB-231 cells (Figure 4D and Figure S1D).

**Figure 5:**
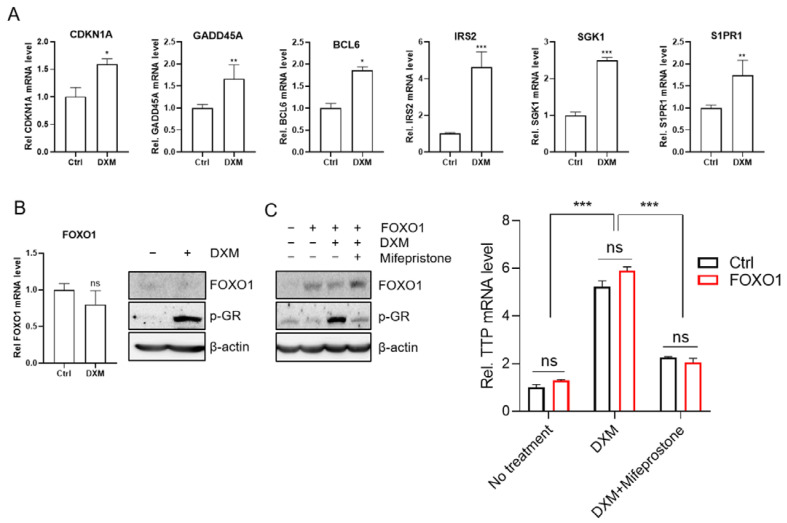
DXM induces *TTP* expression without induction of FOXO1 expression. (**A**) Validation of the DEGs in the FOXO signaling pathway in KEGG analysis by qRT-PCR analysis. MDA-MB-231 cells were stimulated with 500 nM DXM for 3 h. The mRNA expression levels of *CDKN1A*, *BCL6*, *GADD45A*, *S1PR1*, *IRS2*, and *SGK1* were analyzed by qRT-PCR. Fold change in expression level was calculated relative to that of the DMSO control. The graphs are mean ± SD of three independent experiments (Student’s *t* test, * *p* < 0.05; ** *p* < 0.005; *** *p* < 0.001). (**B**) Effect of DXM on the expression of the *FOXO1* in MDA-MB-231 cells. MDA-MB-231 cells were stimulated with 500 nM DXM for 3 h. The expression level of FOXO1 was analyzed by qRT-PCR and western blot analysis. In addition, phosphorylation of GR was determined by western blot. Fold change in expression level was calculated relative to that of the DMSO control. The graphs are mean ± SD of three independent experiments (Student’s *t* test). (**C**) Effect of ectopic expression of *FOXO1* on the DXM-induced expression of TTP in MDA-MB-231 cells. MDA-MB-231 cells were transfected with pFOXO1 plasmid. After 24 h incubation, cells were stimulated with 500 nM DXM in the presence or absence of mifepristone for 3 h. Expression of FOXO1 and phosphorylation of GR were determined by western blot analysis. The expression level of *TTP* mRNA was analyzed by qRT-PCR. Fold change in expression level was calculated relative to that of the DMSO control. The graphs are mean ± SD of three independent experiments (Student’s *t* test and one-way ANOVA, *** *p* < 0.001). ns, not significant.

To determine the involvement of the p53 and Hippo pathways in the induction of *TTP* expression in MDA-MB-231 cells, we treated MDA-MB-231 cells with inhibitors against MDM2 (Idasanutlin) and MST1/2 (XMU-MP-1) to activate the p53 and the Hippo pathways, respectively. Neither inhibitor enhanced the expression of *TTP* in MDA-MB-231 cells, either in the presence of DXM (Figure 4E,F) or BTM-21-P (Figure S1E,F) or in the absence of DXM (Figure 4G). These results, combined with the inhibitor results, suggest that the FOXO1 pathway may mediate the DXM- and BTM-21-phosphate-induced expression of *TTP* in MDA-MB-231 cells. Since both BTM-21-P and DXM induced *TTP* expression in MDA-MB-231 cells through the FOXO1 pathway, we used DXM to induce *TTP* expression in further experiments. qRT-PCR was performed to examine the expression of six genes involved in the FOXO signaling pathway in KEGG analysis: *CDKN1A*, *GADD45A*, *BCL6*, *IRS2*, *SGK1* and *S1PR1*. Consistent with the RNA-seq results, all six genes were significantly increased by DXM treatment in MDA-MB-231 cells (Figure 5A).

The mammalian FOXOs have four members: FOXO1, FOXO3, FOXO4, and FOXO6 [42]. Interestingly, none of the four FOXO members was involved in the upregulated FOXO signaling pathway, and we could not detect them from upregulated DEGs in BTM-21-P and DXM-treated cells (Table S3). qRT-PCR and western blot analyses also confirmed that DXM did not increase the expression of FOXO1 in MDA-MB-231 cells (Figure 5B). However, DXM increased the phosphorylation of GR (Figure 5B). We also found that overexpression of *FOXO1* did not induce the expression of *TTP* in the absence of DXM and did not affect the DXM-induced expression of *TTP* in MDA-MB-231 cells (Figure 5C). In addition, the treatment of cells with a GR inhibitor, mifepristone, blocked the DXM-induced expression of *TTP*, even in *FOXO1*-overexpressing cells (Figure 5C). Collectively, our results suggest that even though DXM induces the expression of *TTP* in a monomeric GR-FOXO1 pathway-dependent manner, the induction of *FOXO1* expression is not required for DXM-induced expression of *TTP*.

The mammalian FOXO family has four members: FOXO1, FOXO3, FOXO4, and FOXO6 [35]. Notably, none of the FOXO members was involved in the upregulated FOXO signaling pathway, and none was not detected from upregulated DEGs in BTM-21-P- and DXM-treated cells (Table S3). qRT-PCR analyses also confirmed that DXM did not increase the expression of *FOXO1* in MDA-MB-231 cells (Figure 5B). We also found that overexpression of *FOXO1* did not induce the expression of *TTP* in the absence of DXM and did not affect the DXM-induced expression of *TTP* in MDA-MB-231 cells (Figure 5C). In addition, the treatment of cells with the GR inhibitor mifepristone blocked the DXM-induced expression of *TTP*, even in *FOXO1*-overexpressing cells (Figure 5C). Collectively, our results suggest that while DXM induced the expression of *TTP* in a monomeric GR-FOXO1 pathway–dependent manner, the induction of *FOXO1* expression is not required for DXM-induced expression of *TTP*.

Inhibition of GR (Figure 2H) or FOXO1 (Figure 4D) abrogated the DXM-induced expression of *TTP*, indicating the requirement for GR and FOXO1 in DXM-induced expression of *TTP* in MDA-MB-231 cells. A search for transcription factor binding sites using online software (JASPAR) revealed the presence of putative FOXO1 binding sites within the *TTP* promoter, but no putative binding sites for FOXO3, FOXO4, FOXO6, and GR were found (Figure S2), indicating that DXM may enhance FOXO1 binding to the *TTP* promoter in a GR-dependent manner in MDA-MB-231 cells.

### 2.5. Dexamethasone-Induced TTP Down-Regulates ARE-Containing Genes in Cancer Cells and Mediates the Anti-Viability Effect of Dexamethasone

**Figure 6:**
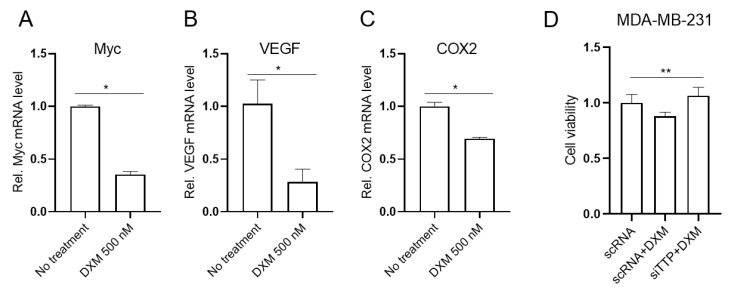
DXM-induced TTP mediates the anti-viability function of DXM in MDA-MB-231 cells. (**A**–**C**) DXM treatment decreases expression of ARE-containing genes in MDA-MB-231 cells. MDA-MB-231 cells were stimulated with 500 nM DXM, and the expression of ARE-containing genes such as (**A**) *Myc*, (**B**) *VEGF*, and (**C**) *COX2* was analyzed by qRT-PCR. Fold change in expression level was calculated relative to that of the DMSO control. The graphs are mean ± SD of three independent experiments (Student’s *t* test, * *p* < 0.05). (**D**) TTP mediates the anti-viability function of DXM in MDA-MB-231 cells. MDA-MB-231 cells were transfected with *TTP*-specific siRNA (siTTP) or control scrambled siRNA (scRNA) and stimulated with 500 nM DXM. Viability of MDA-MB-231 cells was assessed using MTS assays. Graphs show relative cell viability. Data are mean ± SD of three independent experiments (one-way ANOVA, ** *p* < 0.01).

TTP contributes to the down-regulation of ARE-containing genes [1,2]. We next determined whether dexamethasone-induced TTP down-regulated the expression of ARE-containing genes. After treatment with DXM, cells were analyzed for the expression level of TTP-target genes, including *VEGF*, *COX2*, and *Myc* [9,10,12]. DXM treatment led to a decrease in ARE-containing genes in MDA-MB-231 cells (Figure 6A–C).

GCs have been reported to exert anti-tumorigenic effects [43]. To determine whether DXM exhibited anti-viability effects in MDA-MB-231 cells, we incubated cells with 500 nM DXM and analyzed viability using MTS assays. Consistent with previous reports [44,45], DXM (500 nM) significantly inhibited the viability of MDA-MB-231 cells (Figure 6D). TTP exerts anti-viability functions by destabilizing the mRNAs of genes involved in cell viability [4,5]. Thus, we speculated that TTP induced by DXM may mediate the anti-viability effects of DXM in cancer cells. We next tested whether TTP was required for the anti-viability activity of the combined treatment with these compounds. The inhibition of *TTP* using siRNA against *TTP* (TTP-siRNA) attenuated the inhibitory effects of DXM on the viability of MDA-MB-231 cells (Figure 6D). These results suggest that TTP mediates the anti-viability functions of DXM in breast cancer cells.

## 3. Discussion

Approximately 16% of human genes have ARE motifs within their mRNA 3′UTR [46], and many of them are implicated in immune response and tumorigenesis [47,48]. TTP can bind to ARE and enhance the degradation of ARE-containing mRNA by recruiting protein components of P-body to the TTP-mRNA complexes [49,50]. By post-transcriptional down-regulation of the genes involved in tumor onset and progression (4, 5), TTP functions as a tumor suppressor. However, the expression of the TTP tumor suppressor is significantly decreased in various cancers [7]. Thus, inducers of *TTP* in cancer cells with low *TTP* expression may be good candidates for new therapeutic drugs for cancer treatment.

Compounds derived from natural products can demonstrate structural diversity and have the potential to act via diverse mechanisms. Until now, there has been no report of the identification of compounds from a natural compound library that induce TTP expression in cancer cells. The natural compound library provided by the Korea Chemical Bank (http://www.chembank.org/. accessed on 26 January 2017) contains 1019 pure natural compounds from medicinal plants. In this study, we used this library in conjunction with a cell-based reporter assay to discover novel compounds to induce *TTP* expression in cancer cells. By screening the library, we identified one molecule of which the enantiomers are the GCs BTM-21-P and DXM-21-P as a potent inducer of *TTP* in cancer cells. Further characterization revealed that BTM-21-P and DXM-21-P induce *TTP* expression in breast cancer cells in a GR-dependent manner.

Both natural and synthetic GC hormones exert their biological effects predominantly via the GR, a ligand-activated transcription factor that is expressed in nearly all cells [24,25]. DXM, DXM-21-P, and BTM-21-P induced *TTP* expression in a GR-dependent manner in MDA-MB-231 cells. Both monomeric and homodimeric GRs regulate gene expression [27,28,29]. In our study, MDA-MB-231 cells transfected with either wild-type or dimerization-defective GR (GR^dim^) induced similar levels of *TTP* after DXM treatment, indicating that monomeric GR is involved in the DXM-induced expression of *TTP* in MDA-MB-231 cells.

Upon ligand binding, the GR becomes localized in the cell nucleus and binds to DNA sequences called glucocorticoid response elements (GREs) to either inhibit [51,52] or enhance [27,53,54] the expression of numerous genes. However, when predicting transcription factor binding sites, we did not identify GREs within the promoter region of the TTP gene, suggesting that GR may induce the expression of *TTP* in a GRE-independent manner. GR can regulate gene expression without direct binding to GRE by interacting with promoter-bound transcription factors, such as NF-κB, STAT5, and AP-1 [29,30], in a mechanism known as tethering. To better understand the signaling pathways involved in the *TTP* induction in DXM- and BTM-21-P-treated cells, we performed whole genome transcriptome analysis of MDA-MB-231 cells treated with DXM or BTM-21-P for 3 h using RNA-Seq. Both unsupervised hierarchical clustering and DEG clustering in a heat map showed no significant difference between DXM- and BTM-21-P-treated cells, suggesting that they affect similar signaling pathways in MDA-MB-231 cells. A KEGG pathway analysis of up-regulated DEGs in DXM- and BTM-21-P-treated cells revealed upregulation of the FOXO signaling pathway, and inhibition of FOXO1 blocked the DXM- and BTM-21-P-mediated induction of *TTP* expression in MDA-MB-231 cells. In addition, the JASPAR prediction revealed the presence of putative FOXO1 binding sites within the *TTP* promoter. These results suggest that FOXO1 may mediate the effect of GCs on TTP expression in cancer cells. In this study, DXM treatment induced *TTP* expression without increasing FOXO1 level. The over-expression of FOXO1 without DXM stimulation did not enhance TTP expression in MDA-MB-231 cells. Collectively, these results suggest that the GC/GR signal pathway does not increase *FOXO1* expression but may increase FOXO1 binding to the *TTP* promoter, which leads to induction of *TTP* expression in MDA-MB-231 cells.

GCs have been reported to inhibit the growth of cells [43,44,45], and TTP also inhibits the growth of cancer cells by down-regulation of ARE-containing genes involved in cell proliferation [9,10,12]. In our study, *TTP* expression was induced in MDA-MB-231 cells. This suggests that GC-induced *TTP* may inhibit the growth of cancer cells through down-regulation of ARE-containing genes. Indeed, GC treatment down-regulated the expression of ARE-containing TTP target genes such as *Myc*, *VEGF*, and *COX-2* and inhibited the viability of MDA-MB-231 cells. Inhibition of *TTP* by siRNA against *TTP* attenuated DXM-induced inhibition of cell viability, indicating that DXM-induced TTP plays a role in the anti-viability effect of DXM. Even though we found that GCs increased the TTP expression and inhibited viability of MDA-MB-231 cells, it is not likely that all kinds of breast cancer cells will show a similar response to GCs. GCs can promote metastasis of certain type of breast cancer cells [55,56], and TTP inhibits the migration of cancer cells by suppressing the expression of *Twist1* and *Snail1* [57]. Further study is required to determine whether GCs induces TTP expression in breast cancer cells in which metastasis is promoted by GCs.

## 4. Materials and Methods

### 4.1. Cells and Chemicals

The human MCF-7, MDA-MB-231, HCC-1143, HCC-1187, BT20, BT-474, and T47D breast cancer cell lines were purchased from the Korean Cell Line Bank (Seoul, Korea). Cells were cultured in RPMI 1640 media supplemented with 10% heat-inactivated fetal bovine serum (FBS) (Welgene, Korea) and were maintained at 37 °C in a humidified 5% CO_2_ atmosphere. Dexamethasone (DXM), dexamethasone 21-phosphate (DXM-21-P), betamethasone 21-phosphate (BTM-21-P), mifepristone (Sigma Aldrich, St. Louis, MO, USA), Wortmannin, QNZ, MK2206, idasanutlin, AS1842856, XMU-MP-1 and p38 MAPK inhibitor (Selleckchem, Houston, TX, USA) were used in this study.

### 4.2. Cell Viability

For the MTS assay, cells were plated in triplicate at 1.0 × 10^4^ cells/well in 96-well culture plates in culture media. At 24 h after plating, CellTiter 96 AQueous One Solution reagent (Promega, Madison, WI, USA) was added to each well according to the manufacturer’s instructions, and absorbance at 490 nm was determined for each well using a Victor 1420 Multilabel Counter (EG&G Wallac, Turku, Finland).

### 4.3. Plasmids, Small Interfering RNAs, Transfections, and Dual-Luciferase Assay

The pGL3/TTPp-1343 plasmid containing the human *TTP* promoter was described previously [11]. The pGR-wt, pGR^dim^, and pFOXO1-Flag plasmids were purchased from Addgene (Watertown, MA, USA).

Small interfering RNAs (siRNAs) against human *TTP* (TTP-siRNA, sc-36761) and control siRNA [scrambled siRNA (scRNA), sc-37007] were purchased from Santa Cruz Biotechnology (Santa Cruz, Santa Cruz, CA, USA). Cells were transfected 24 h after plating using LipofectamineTM RNAiMAX (Invitrogen, Carlsbad, CA, USA) and harvested at 48 h after transfection. The expression levels of *TTP* mRNA were analyzed by qRT-PCR.

### 4.4. Screening of the Natural Product Library and Luciferase Assay

Natural compounds have been used to develop drugs for cancer and infectious diseases, since they are structurally optimized by evolution to serve particular biological functions, and their use in traditional medicine provides insights regarding efficacy and safety [58]. Thus, in this study, we used a natural product library to select natural compounds which can induce TTP expression in cancer cells. A library containing 1019 natural products was provided by the Korea Chemical Bank (http://www.chembank.org/. accessed on 26 January 2017) of the Korea Research Institute of Chemical Technology. MCF-7 cells in culture dishes (100 mm diameter) were co-transfected with the pGL3/TTPp-1343-luciferase reporter construct and pRL-SV40 Renilla luciferase construct using TurboFectTM in vitro transfection reagent (Fermentas, Waltham, MA, USA). After incubation for 24 h, cells were harvested and seeded in 96-well plates at a density of 4 × 10^3^ cells per well in 100 μL and cultured with 30 μL of natural compounds diluted in fresh culture media. After further incubation for 24 h, cells were lysed with lysis buffer and mixed with luciferase assay reagent (Promega, Madison, WI, USA). Cells were also treated with the same volume of DMSO to detect luciferase activity induced by the native signal pathway. The chemiluminescent signal was measured using a SpectraMax L Microplate (Molecular Devices, Sunnyvale, CA, USA). Firefly luciferase was normalized to Renilla luciferase in each sample. All luciferase assays reported in this study represent at least three independent experiments, each consisting of three wells per transfection. We selected compounds that induced a greater than two-fold increase in luciferase activity.

### 4.5. Quantitative Real-Time PCR and Semi-qRT-PCR

DNase I–treated total RNA (3 μg) was reverse transcribed using oligo-dT and Superscript II reverse transcriptase (Invitrogen, Carlsbad, CA, USA) according to the manufacturer’s instructions. Quantitative real-time PCR (qRT-PCR) was performed by monitoring in real-time the increase in fluorescence of SYBR Green dye (QIAGEN, Hilden, Germany) using the StepOnePlus^TM^ real-time PCR system (Applied Biosystems, Waltham, MA, USA). Semi-qRT-PCR was performed using Taq polymerase (Solgent, Daejeon, Korea) and the PCR primer pairs (Table 1).

### 4.6. SDS-PAGE and Immunoblotting

Proteins were resolved by SDS-PAGE and transferred onto Hybond-P membranes (Amersham Biosciences Inc., Amersham, UK). The membranes were blocked and then probed with appropriate dilutions of the following antibodies: rabbit anti-human TTP (T5327, Sigma, St. Louis, MO, USA) and anti-β-actin (A2228, Sigma, St. Louis, MO, USA). Immunoreactivity was detected using an ECL detection system (Amersham Biosciences Inc., Amersham, UK). Films were exposed at multiple time points to ensure that the images were not saturated.

### 4.7. RNA Preparation and RNA-Seq

We performed RNA-Seq on total RNA samples (RIN above 8.5) collected from MDA-MB-231 cells at 4 h after treatment with growth media control, 500 nM DXM-21-P or 500 nM BTM-21-P. Residual DNA from each sample was removed using the RNeasyMinEluteCleanup Kit (Qiagen, Hilden, Germany). The cDNA library was prepared with 1.0 μg of total RNA using the TrueSeq RNA library Preparation Kit (Illumina, San Diego, CA, USA) following manufacturer’s recommendations, followed by paired-end sequencing (2 × 100 bp) using the HiSeq1500 platform (Illumina, San Diego, CA, USA). cDNAs were amplified according to the RNAseq protocol provided by Illumina and sequenced using an Illumina HiSeq 2500 system to obtain 150-bp paired-end reads. The sequencing depth for each sample was >20 million reads. RNA-seq reads were mapped using STAR 2.7.9a [59] to the human genome GRCh38. Gene expression counts were measured using multicov implemented in bedtools [60]. Differentially expressed genes (DEGs) were obtained by comparing groups (Control, Beta, and Dexa) using EdgeR [41]. Genes with false discovery rate (FDR) <0.01 and log2fold change >0.3 were selected as DEGs. The DEGs were clustered using hierarchical clustering implemented in R. Ward’s criterion. Pearson’s correlation coefficient was used as a distance measure. A clustering heatmap was drawn using a z-score scaled across samples for each gene. The enriched KEGG pathway terms were obtained from Enrichr software [61].

### 4.8. Statistical Analysis

Differences in the expression of genes were evaluated by Student’s t-test or one-way ANOVA. A *p* value less than 0.05 was considered statistically significant.

## 5. Conclusions

Herein, we have reported that GC compound, an enantiomer of BTM-21-P and DXM-21-P, identified from a natural compound library, induces *TTP* expression in a GR-dependent manner in breast cancer cells. Furthermore, we found that GR does not induce *FOXO1* expression but may stimulate FOXO1 to bind to the TTP promoter and thus to induce TTP expression. Importantly, GC-induced TTP down-regulated ARE-containing TTP target genes and mediated the anti-viability function of GCs. The inhibition of *TTP* by siRNA attenuated the anti-viability effect of GCs. Thus, our data indicate that GCs induce *TTP* expression in a FOXO1-dependent manner, and that GC-induced TTP mediates the anti-viability activity of GCs in breast cancer cells.

## Figures and Tables

**Table 1:** PCR primers used in this study.

Genes			Primer Sequences (5′-3′)
Gene expression analysis	*β-actin*	F	ATCGTGCGTGACATTAAGGAGAAG
		R	AGGAAGGAAGGCTGCAAG
	*BCL6*	F	CATGCAGAGATGTGCCTCCACA
		R	TCAGAGAAGCGGCAGTCACACT
	*CDKN1A*	F	AGGTGGACCTGGAGACTCTCAG
		R	TCCTCTTGGAGAAGATCAGCCG
	*FOXO1*	F	CTACGAGTGGATGGTCAAGAGC
		R	CCAGTTCCTTCATTCTGCACACG
	*GADD45A*	F	CTGGAGGAAGTGCTCAGCAAAG
		R	AGAGCCACATCTCTGTCGTCGT
	*GAPDH*	F	AATCCCATCACCATCTTCCAG
		R	AAATGAGCCCCAGCCTTC
	*IRS2*	F	CCTGCCCCCTGCCAACACCT
		R	TGTGACATCCTGGTGATAAAGCC
	*S1PR1*	F	CCTGTGACATCCTCTTCAGAGC
		R	CACTTGCAGCAGGACATGATCC
	*SGK1*	F	GCTGAAATAGCCAGTGCCTTGG
		R	GTTCTCCTTGCAGAGTCCGAAG
	*TTP*	F	TCTTCGAGGCGGGAGTTTTT
		R	TGCGATTGAAGATGGGGAGTC

## Supplementary Materials

The following supporting information can be downloaded at: https://www.mdpi.com/article/10.3390/ijms232213673/s1.
