# Supplementary material for: FOXO1 Is a Key Mediator of Glucocorticoid-Induced Expression of Tristetraprolin in MDA-MB-231 Breast Cancer Cells
Source: Int J Mol Sci. 2022 Nov 8;23(22):13673. doi: 10.3390/ijms232213673 (PMC9693238; doi:10.3390/ijms232213673)
Supplement: Supplementary file 1 [file ijms-23-13673-s001.zip › Supplementary Figures.pdf]

FOXO1 Is a Key Mediator for Glucocorticoid-Induced Expression of Tristetraprolin in MDA-MB-231 Breast Cancer Cells

Do Yong Jeon[1,⁋], So Yeon Jeong[1,⁋], Ju Won Lee[1,⁋], Jeonghwan Kim[2], Jee Hyun Kim[3], Hun So Chu[3], Won Jin Jeong[3], Byung Ju Lee[1], Byungyong Ahn[4], Junil Kim[2], Seong Hee Choi[3,*], Jeong Woo Park[1,*]

1 Department of Biological Sciences, University of Ulsan, Ulsan 44610, Korea

2 School of System Biomedical Science, Soongsil University, Seoul 06978, Korea

3 RopheLBio, B102, Seoul Forest M Tower, Seoul 04778, Korea

4 Department of Food Science and Nutrition, University of Ulsan, Ulsan 44610, Korea

* Correspondence: Seong Hee Choi nacchal@ulsan.ac.kr; Jeong Woo Park jwpark@ulsan.ac.kr

⁋These authors contributed equally to this work.

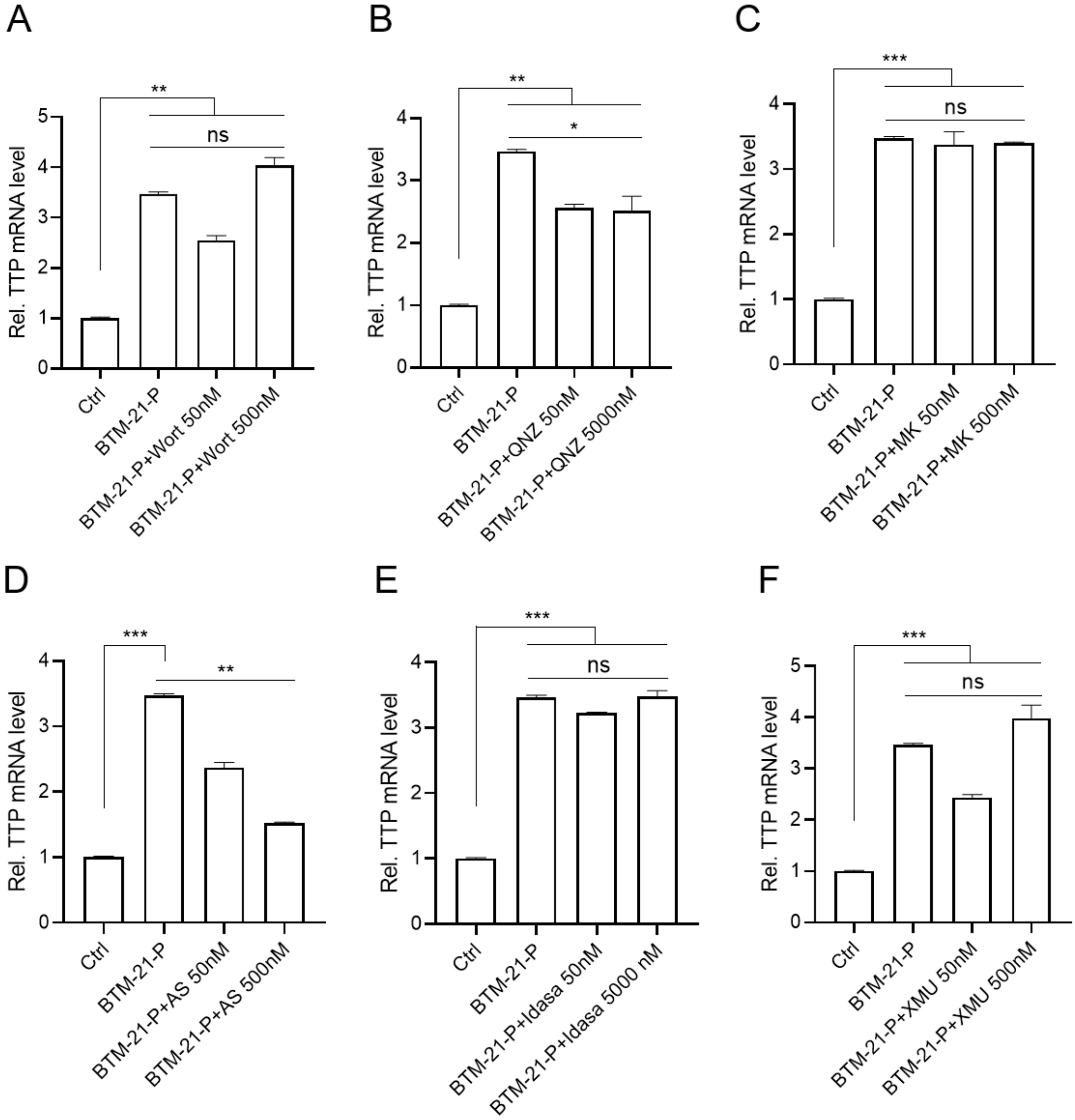

Supplement Figure S1. FOXO1 mediates BTM-21-P-induced TTP expression in MDA-MB-231 cells. MDA-MB-231 cells were incubated in the presence of indicated concentration of inhibitors against (A) PI3K (Wortmannin) (B) NF-κB (QNZ), (C) AKT (MK2206), (D) FOXO1 (AS1842856), (E) MDM2 (Idasanutlin), and (F) MST1/2 (XMU-MP-1) for 12 h, followed by stimulation with 500 nM BTM-21-P for 3 h. The expression level of TTP was analyzed by qRT-PCR. Fold change in expression level was calculated relative to that of DMSO control. The graphs are means ± SD of 3 independent experiment. (One-way ANOVA, *, $p<0.05$; **, $p<0.005$; ***, $p<0.001$). ns, not significant.

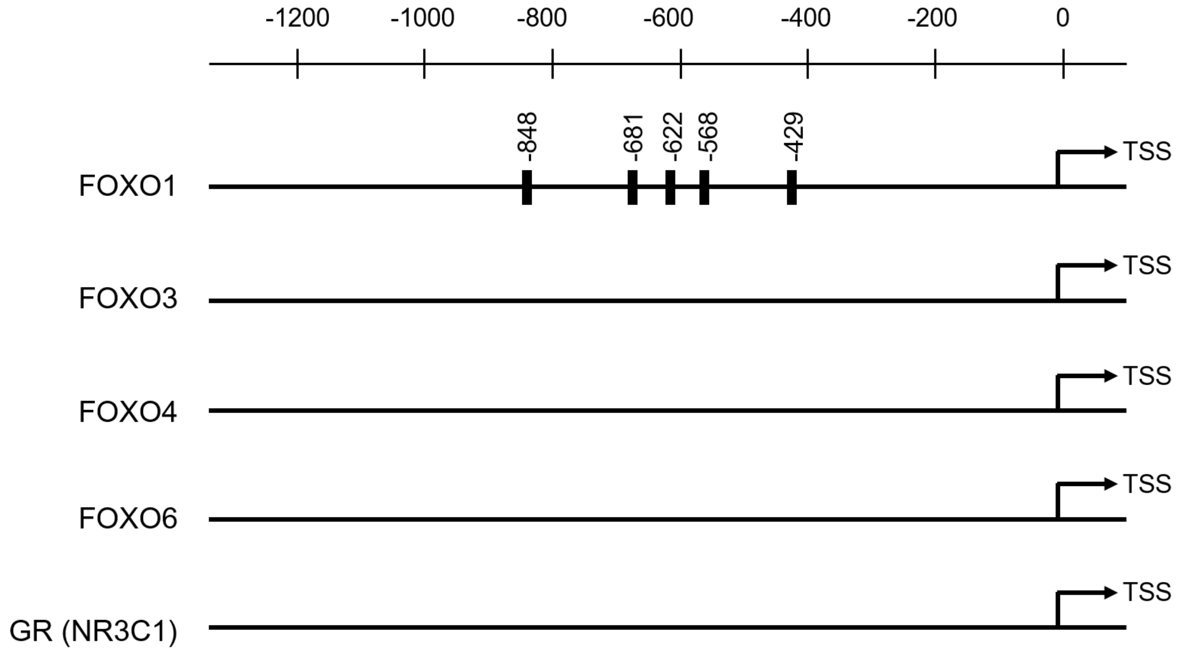

Supplementary Figure S2. The JASPAR prediction (*p* value = 0.001) for the binding positions of FOXO1, FOXO3, FOXO4, FOXO6, and GR (NR3C1) on the promoter of the TTP gene. TSS, transcription start site.
