# Supplementary material for: FOXO1 Is a Key Mediator of Glucocorticoid-Induced Expression of Tristetraprolin in MDA-MB-231 Breast Cancer Cells
Source: Int J Mol Sci. 2022 Nov 8;23(22):13673. doi: 10.3390/ijms232213673 (PMC9693238; doi:10.3390/ijms232213673)
Supplement: Supplementary file 1 [file ijms-23-13673-s001.zip › Table S2. Fold changes in TTP promoter activity by 5 selected compounds.pdf]

Table S2. Fold changes in TTP promoter activity induced in breast cancer cells by 5 selected natural compounds.

Compound name	Fold changes in TTP-Promoter Luciferase activity						
	1st screening (30µl)	2nd screening (30µl)	3rd screening (30µl)	4th screening			
				5µl	10µl	30µl	50µl
01-E10	5.12	2.09	3.27	3.63	4.77	6.82	6.37
01-G05	5.28	2.52	2.4	1.49	2.02	4.21	5.50
07-B10	3.29	2.43	2.61	1.18	1.32	1.28	1.66
07-A10	2.99	2.04	2.38	1.44	1.12	1.49	1.56
05-A06	2.32	2.69	3.14	2.45	3.28	5.64	5.91

1st, 2nd, and 3rd screenings were conducted using MCF-7 cells and 4th screening was conducted using MDA-MB-231 cells.
